# Poor quality male latex condoms found in Dominican Republic: Quality assurance evaluation and public health impact

**DOI:** 10.1371/journal.pone.0210150

**Published:** 2019-01-07

**Authors:** Jeff Tremelling, Allen All, Luis Lleras, Aida Cancel, David Jenkins, Carolina Pina, Damani Goldstein, Clancy Broxton, Steve Hamel

**Affiliations:** 1 Product Quality and Compliance, FHI 360, Durham, NC, United States of America; 2 United States Agency for International Development, Santo Domingo, Dominican Republic; 3 United States Agency for International Development, Washington, DC, United States of America; US Centers for Disease Control and Prevention, Dengue Branch, PUERTO RICO

## Abstract

Male condoms are important to prevent transmission of HIV (and other STIs) and unwanted pregnancies. Research was conducted to evaluate the quality of male condoms available in the Dominican Republic market based on preliminary concerns of suspect product. Based on international testing standards (ISO 4074 and ASTM D 3492–08), condoms were sampled across ten prominent brands within the market and evaluated for airburst pressure / volume, freedom from holes, visual defects, package seal integrity, packaging and marking, lubricant quantity, and dimensions. Five of the brands were found to have extensive quality problems, where holes were found in 5.7% to 17.5% of the condoms (depending on the brand). Between 5.1% and 30.5% of these condoms failed to meet the requirements for airburst properties, and violations in regulatory labeling where observed. Three additional brands were compliant for the other tests, but were found to have the same challenges with labeling violations as the previous five brands. Two brands were found to be fully compliant with all aspects of the evaluation. The level of defects observed in these samples would greatly increase the risk of HIV transmission (and other STIs) and unwanted pregnancies. When projected on the annual market of male condoms sold in the Dominican Republic (~26 million), potentially over 1 million condoms could be estimated to adversely impact the health risk of the end-user. These results prompted action by the Dominican Republic regulatory authorities to investigate and remove poor quality product from the market. This research study emphasizes the need for continued vigilance towards increased regulatory and market surveillance efforts to better protect public health interests.

## Introduction

Poor quality products for global public health present an enormous risk to the end user. Examples of poor quality pharmaceuticals in the market may be the most prominent type reported (especially for anti-malarials) [1–10], but other product types can be at risk for quality issues. Specifically for condoms, concerns regarding quality have been reported in Ethiopia [11], Vietnam [12], India [13], and China [14], where problems ranged from holes in the condoms, rupture during use, and falsified branding.

Male latex condoms are a key commodity to prevent unintended pregnancies and transmission of human immunodeficiency virus (HIV) and other sexually transmitted infections (STIs) [15]. Billions of condoms are manufactured and procured annually, and it was estimated in 2015 that 27 billion condoms were globally available to provide approximately 225 million couple years protection (level of protection provided over a one year period) from unintended pregnancies and significantly reduced rates of HIV and other STIs [16]. In the Dominican Republic, high quality condoms are needed to prevent continued transmission of HIV, STIs, and reduction in unintended pregnancies. With 66,000 people currently living with HIV in the Dominican Republic, sustained efforts to control transmission remain important. Condoms remain a cornerstone of the HIV prevention effort, as well as an important contraceptive method, with 67.4% of young men who have multiple partners reporting use of a condom during their last sexual intercourse [17].

Male condoms are a regulated commodity in most countries requiring approval from a national (or regional) regulatory authority or ministry of health prior to market use to help ensure that these meet technical and quality standards. International standards (i.e., ISO 4074 [18] and ISO 13485 [19]) are used to regulate condom production to ensure acceptable quality standards are met.

Here we report the outcome of research in the Dominican Republic that began as part of a preliminary distribution check (through the Ministry of Health) where poor quality condom brands were suspected to be available in ~60% of grocery stores and ~40% of pharmacies within the general Dominican Republic market. The United States Agency for International Development (USAID) mission office in the Dominican Republic requested an investigation of the quality of multiple condom brands available in the country. USAID, with support of the Ministry of Health, engaged FHI 360 for quality assurance testing in order to ascertain potential health risks.

## Materials and methods

Condom brands were chosen for testing based upon an initial visual review of the product and packaging of a small sample of each brand, yielding eight condom brands suspected to have quality issues. Two additional brands were sampled for testing as controls that originated from known high-quality manufacturers that performed well on pre-shipment testing prior to arrival in-country. After consultation and approval from the Ministry of Health and the Dirección General de Drogas y Farmacias, USAID/Dominican Republic sampled from various locations in the greater Santo Domingo area and shipped a total of ten condom brands (720 condoms for brands 1–7 and 9–10; brand 8 had 360 condoms) for testing. For each brand, samples were from the same lot. All condom brands chosen for testing were readily available in the Dominican Republic at the time of testing. Specific manufacturer and product brand names are not disclosed, but rather sampled brands are identified with a numerical code. All samples obtained were well within the listed expiration date at the time of testing.

All tests were conducted based on ISO 4074 procedures [18], where the number of samples tested were based on requirements in ISO 4074:2002 [18a], ASTM D3492-8 [20], and ISO 2859–1 [21] sampling procedure for inspection by attributes based on lot size. Although actual lot sizes for each brand was unknown, sample sizes (Table 1) were based on our knowledge and experience of common lot sizes and are expected to provide a high degree of confidence in the results.

**Table 1:** ISO 4074 test results for sampled brands of male latex condoms from the Dominican Republic.^a^ Except for the average lubricant quantity (mg), the values presented for each brand / test combination represent the number of failing samples observed.

Test^b^	Airburst Pressure / Volume	Freedom from Holes	Visible Defects	Average Lubricant (mg)	Package Seal	Dimensions	Packaging and Marking	Lot Recommendation
Number of Condom Samples Tested per Brand	315	315	315	13	32	13	32	
Accept / Reject Tolerances^c^	10/11	2/3	3/4	n/a	2/3	0/1	2/3	
**Poor Quality / Serious Health Risk**								
Brand 1	**16**	**18**	3	261	0	0	**32**	**Non-Compliant**
Brand 2	**96**	**21**	**5**	238	2	0	**32**	**Non-Compliant**
Brand 3	**45**	**44**	1	249	0	0	**32**	**Non-Compliant**
Brand 4	**20**	**55**	1	293	0	0	**32**	**Non-Compliant**
Brand 5	**27**	**46**	0	266	0	0	**32**	**Non-Compliant**
**Regulatory Labeling Violations**								
Brand 6	4	0	0	245	0	0	**32**	**Non-Compliant**
Brand 7	3	1	0	256	0	0	**32**	**Non-Compliant**
Brand 8^d^	4	0	0	212	0	0	**20**	**Non-Compliant**
**Acceptable Quality**								
Brand 9	1	0	0	520	0	0	0	Compliant
Brand 10	2	0	0	521	0	0	0	Compliant

a–Bold font indicates brands and test results that did not meet ISO 4074:2002. Each set of samples was from the same lot for each respective brand.

b–Test results presented as the number of failures observed for the amount tested per lot.

c–The accept / reject (A/R) tolerances indicates the maximum number of failures that are acceptable and the minimum number of failures that reject the lot, respectively.

d–Because of limited sample, the following number of samples and A/R were utilized for the listed tests; Airburst (125, 5/6), Freedom from Holes (200, 1/2), Visible Defects (200, 2/3), Avg. Lubricant (13, 400–700 mg range), Package Seal (20, 1/2), Dimensions (13, 0/1), Packaging and Marking (20, 1/2). ISO 4074:2002 and ISO 2859–1 have provisions for smaller sample sizes with adjustments in A/R levels, allowing for interpretation of data from different sample sizes with the same acceptance quality limit. It is acknowledged that the probabilities of observing non-compliant lots can be different depending on the amount sampled[21].

### Airburst pressure and volume

Airburst followed ISO 4074:2002 [18a] (section 6.1 and Annex G) using Aladan instruments designed to meet ISO 4074 standard requirements. The inflation length of the condom was 150 ± 3 mm and the test mandrel had a hemispherical diameter of 25 mm. The machine delivered clean (oil/moisture-free) air at a rate of 24–30 dm^3^/min, verified daily with an annually calibrated flow-meter (Sierra Instruments–Model 822-13-OV1-PV1-V1). The pressure was measured with annually calibrated transducers (Prosense–Model PTD25-20-0100WCH) in kilopascals (to the nearest 0.1 kPa). Condoms with widths ≤ 50.0 mm must have a minimum airburst volume of 16.0 dm^3^, and condoms with widths 50.0–56.0 mm must have a minimum airburst volume of 18.0 dm^3^. The burst pressure must be a minimum of 1.0 kPa regardless of width. Unless stated otherwise, 315 condom samples were tested for each brand.

### Visual defects

Testing followed ISO 4074:2002 [18a] (section 9, Annex L.2.3.3, and L.3.3.4). Condoms selected for the freedom from holes (FFH) test were first subjected to the visible defects test. Upon removing from the condom package, they were unrolled and inspected for visible holes, tears and other visible defects as described in the applicable standards and specifications. If visible holes or tears were observed, the condom was considered non-compliant and further tests were halted (for that condom). Other visible defects were recorded and the condom then was subjected to the FFH test. Unless stated otherwise, 315 condom samples were evaluated for each brand.

### Freedom from holes (FFH)

Testing followed ASTM D3492-8 Section A3. Equipment used was non-branded and built specifically to meet the FFH requirements to deliver 300 ± 10 ml of water with a temperature between 10°C and 40°C. The test mounts were free of defects that may damage and allowed the condom to hang freely from the machine. Samples were filled with water and allowed to hang for at least one minute. Each was manipulated by hand pushing water into the closed end, middle and top of the condoms while examining for leaks. If leakage was noted, the condom was marked and measured. Condoms identified with holes greater than 25 mm from the open end were recorded as non-compliant. Unless stated otherwise, 315 condom samples were tested for each brand after previously being evaluated for visible defects.

### Package seal integrity

Testing followed ISO 4074:2002 [18a] (section 10 and Annex M). The equipment consists of a plastic vacuum bell jar (Nalgene Amber PEI) capable of withstanding the required vacuum of 20 ± 5 kPa absolute pressure, a venturi vacuum pump (Cole Parmer Venturi-Style Model 78165–20) capable of obtaining the required pressure, compressed air source, calibrated vacuum gauge (Marshalltown 89998), clamping device and degassed water covering the test samples by no less than 25mm. The vacuum was applied to the samples for 1 minute. If a bubble stream was observed from a package, it was determined to be non-compliant. Each package was opened after removal form the vacuum chamber. If water was found inside the package, it was determined to be non-compliant. Unless stated otherwise, 32 condom samples were evaluated for each brand.

### Packaging and marking

Testing followed ISO 4074:2002 [18a] section 11. If any packing or marking requirements were not met, the condom was recorded as non-compliant. In addition, the retail, consumer, and primary packaging of all samples were reviewed in accordance with the Dominican Republic Ministry of Health, Dirección General de Drogas y Farmacias regulatory labeling requirements [22]. Unless stated otherwise, 32 condom samples were evaluated for each brand.

### Lubricant quantity

ISO 4074:2014 [18b] Annex C was followed which allows propan-2-ol or an aqueous surfactant for removal of silicone lubricant from the condom. From internal data, the two methods were compared for silicone lubricant removal using the *h* and *k* statistics used in ASTM E-691. Two different condom lots (each from a different manufacturer) were evaluated with two replicates from the more traditional propan-2-ol and four replicates with the aqueous surfactant method using 0.5% Dawn Professional Detergent, Procter & Gamble (50 ml of detergent diluted to 10 L). Comparing the data from both methods found no outliers from any of the replicates, where the overall measurement uncertainity was calculated to be ± 20 mg of silicone lubricant. The laboratory was transitioning from propan-2-ol to the aqueous surfactant. Some lots were tested with propan-2-ol and others with aqueous surfactant. Brands 1, 4, 6, and 10 were tested with the propan-2-ol and brands 2, 3, 5, 7, 8, 9 were tested with the aqueous surfactant method. Each condom package was weighed using a calibrated balance (AdventurePro AV213C) accurate to 1 mg. Three sides of the condom package were opened using a scalpel, the condom was removed, cut with a pair of scissors from the end to the center and then unrolled. The condom and package were then washed with the cleaning solution and dried in a calibrated oven for at least 15 minutes for propan-2-ol and 30 minutes for aqueous surfactant. The condom and package were then reweighed. The difference between the initial weight and second weight was recorded as the lubricant quantity. ISO 4074 [18] does not include a lubricant quantity specification. WHO/UNFPA specifications [15] recommend silicone lubricant levels of 400–700 mg (commonly applied to public markets), but lubricant levels outside of this range (i.e., at lower levels) can be approved in commercial markets. Unless stated otherwise, 13 condom samples were tested for each brand.

### Dimensional tests

Testing followed ISO 4074:2002 [18a] section 5.3 (Annex D-F) with ASTM D3492-8 [20] section 4.2.1.3. Length testing was done after removing the lubricant from whole condoms and drying the condom. The condom was allowed to hang freely over a calibrated graduated mandrel with a spherical radius of 12.5 mm, followed by recording the shortest length where any length less than 160 mm was non-compliant. Using the same condom, width was determined by placing the condom over a calibrated steel ruler at the narrowest part of the condom which is 35 mm or less from the open end. From the stated nominal width, any measurement more than ±2 mm was considered non-compliant. Using the same condom, thickness was measured using the micrometer method (Annex F and ASTM D3492-8 section 4.2.1.3). The thickness (double layer with condom laying flat) was taken at three points along the length (30 ± 5 mm, 90 ± 5 mm, and 150 ± 5 mm) using an ASTM D3767 [23] compliant thickness gauge with a foot pressure of 22 ± 5 kPa. The results were averaged and divided by two to arrive at the single wall thickness. ISO 4074 [18] does not include a thickness specification. WHO/UNFPA specifications [15] recommend a thickness range of 0.045–0.080 mm (commonly applied to public markets), but thicknesses outside of this range can be approved in commercial markets. Unless stated otherwise, 13 condom samples were tested for each brand after previously being evaluated for lubricant quantity.

## Results

Test results for the ten condom brands sampled are provided in Table 1. Based on the test results, all samples were then categorized into three distinct quality levels:”poor quality / serious health risk,”“regulatory labeling violations,” and”acceptable quality.”

### Poor quality / Serious health risk

Sampled brands 1–5 failed to meet requirements for airburst pressure/volume, FFH, and packaging and marking. Furthermore, samples from brand 2 did not meet the visible defects standard. For these five brands, between 5.1% and 30.5% of the condoms tested failed to meet the minimum burst property requirements and are at risk of failing in use, and between 5.7% and 17.5% of the condoms tested had holes. Condoms with the number of quality defects observed during testing would increase the health risk to consumers (assuming a direct correlation with risk and defect level).

Regarding packaging, brands 1–5 were missing the required manufacturer/title holder name information, where brands 4 and 5 did not include an expiration date or lot number on the outer box (inner foil packages did have expiration date and lot number). Brands 1, 2, 4, and 5 did not include a registration number on the packaging. The validity of the registration number from brand 3 was unable to be confirmed from available information.

### Regulatory labeling violations

Although the quality differences of brands 6–8 were not as serious as the previous five brands (with compliant results for airburst and FFH), sufficient packing and marking information (including registration number) was not present based on international standards. Packaging for brands 6 and 7 did not include manufacturer/title holder name and contact information. Brand 6 did not include a lot number or an expiration date on the packaging.

### Acceptable quality

There were no quality concerns raised with brands 9 and 10 because all international standard and regulatory requirements were met for these samples [15,17,22]. These brands are commonly supplied by various donor agencies, such as USAID and UNFPA.

### Other product observations

Lubricant levels of brands 1–8 were lower than brands 9 and 10. While all of these levels may be compliant with market regulations, the range of lubricant observed may provide noticeable differences during intercourse depending on user preferences. WHO/UNFPA specifications indicate that condoms should be packaged in square or circular packages so the rolled condom is not distorted from the circular shape [15]. Brands 1–8 were in rectangular packages which cause the rolled condom to bend inside the package, thus distorting the condoms. It should be noted that condoms in rectangular packages are available in some markets [24]. Changes in physical properties of condoms may potentially be observed with different packaging shapes during storage, but the level of impact should be evaluated for the product source and packaging material used [25]. Brands 1–6 and 8 claimed that the condoms were scented, namely chocolate, mint, orange, strawberry, grape, apple and fruit. Scents were difficult to detect, non-existent, or did not match the description. Additionally, each scent could have a unique lot number for easier traceability. Each brand only had one lot number without assigning a different sub-lot number for scent.

Brands 2–4, 6 and 7 claimed to be dotted condoms, but these features were inconsistent in terms of visualizing and feeling the dots. The ribbed features claimed on brand 5 were inconsistent, with some samples having easily identified ribs (could see and feel the ribs) and other samples could be seen but not felt. Labeling from brand 3 could be interpreted as having condoms larger than average size, but was not substantiated from length and width measurements. Brands 4 and 5 had identical lot numbers, which would be rare if manufactured by two different companies as each company would have a unique assignment method. Brands 2–8 did not contain detailed storage information and brand 3 did not provide disposal information.

**Fig 1:**
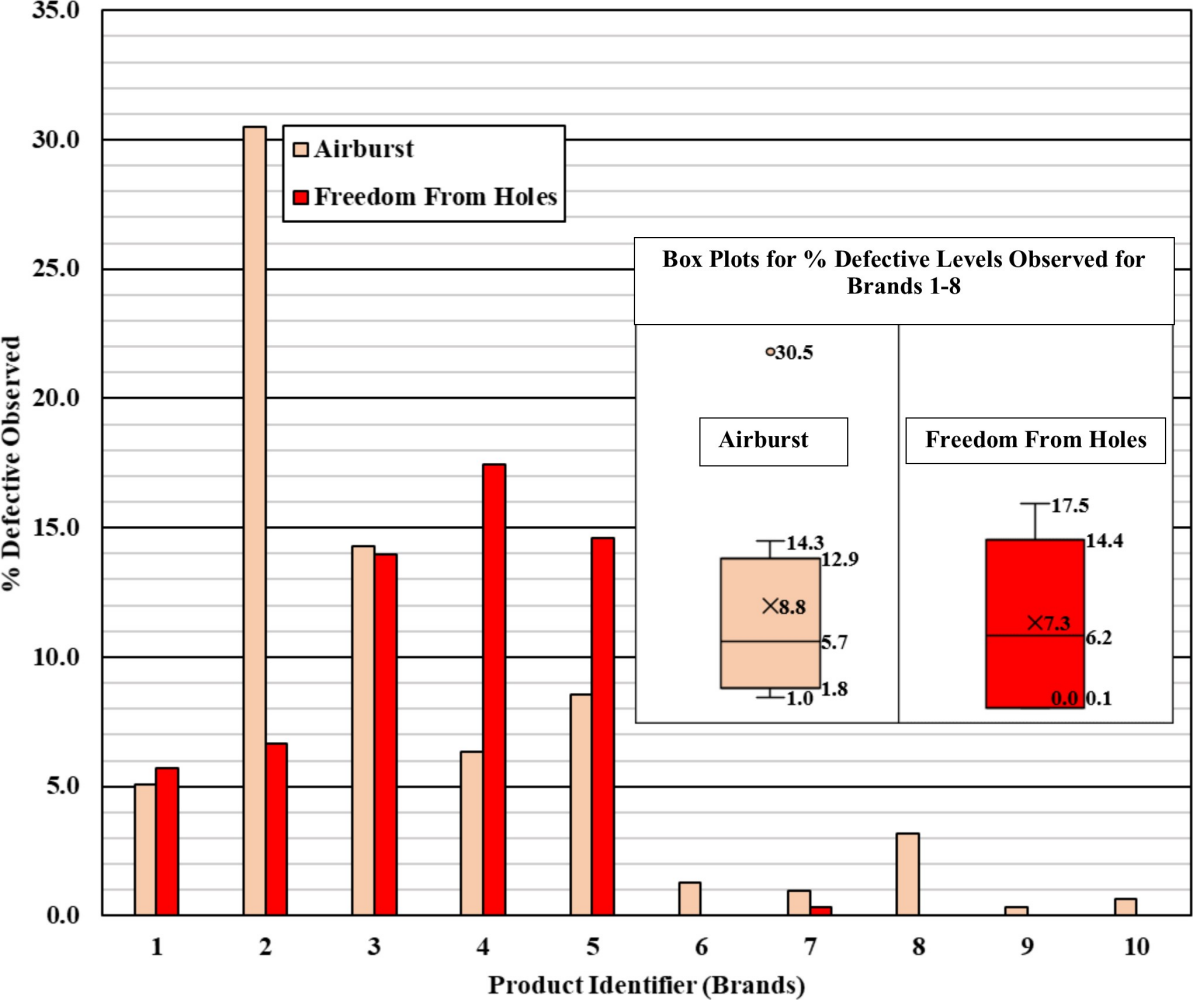
Percent defective (based on number of failures found relative to the number tested as shown in Table 1) observed for both airburst and freedom from holes testing for each sampled brand.

The test results from this study indicate a high number of poor quality condoms in the market. The most concerning results were the high number of defects for airburst and FFH observed for brands 1–5. Although brands 1–8 had low lubricant levels and non-compliant packaging/marking, problems with airburst and FFH will present the greatest risk to the end-user because the barrier characteristics have a higher likelihood of being compromised. Based on the results from Table 1, percent defectives were calculated for each of the brands for both airburst and FFH testing, where sampled brands 9 and 10 are presented as control data (Fig 1). Across brands 1–8, the overall average percent defective observed was 8.8% (interquartile range of 1.8% - 12.9%) and 7.3% (interquartile range of 0.1% to 14.4%) for airburst pressure/volume and FFH, respectively.

## Discussion

The clinical effectiveness of male condoms is dependent on the product and the user. For contraception, approximately 2% of couples using male condoms could become pregnant with consistent / correct use during the first year, while increasing to 18% with typical use [26]. For preventing the transmission of sexually transmitted infections (STIs), consistent use of condoms can provide an efficacy ranging from ~80–95%, depending on the specific STI and route of transmission [26].

The high level of holes found in the condoms inherently demonstrates a comprised barrier. Non-compliant results for airburst is an indication that the latex material does not have an appropriate level of physical strength and homogeneity, thus increasing breakage risk during use [17]. Furthermore, low lubricant levels could present a different user perception during intercourse. Although it is difficult to quantify the impact, the number of defects observed would substantially decrease the overall expected efficacy experienced by the user for preventing unwanted pregnancies and STI transmission.

Approximately 26 million condoms are sold annually in the Dominican Republic, according to a condom use market analysis [27]. One of the control brands in our study (within brands 9 and 10) represents 48% of the market share in the Dominican Republic [27], while the market share of the other control brand could be considered negligible. The overall percent defective for brands 9 and 10 was estimated at 0.5% for airburst, and no holes were found in any of the condoms tested. Thus, if we consider a condom quality comprised of a ≤ 0.5% defect level (for both defect types) on this portion of the market, then we can estimate approximately 12.5 million condoms offer adequate end user protection.

Our data suggest that several brands in the remaining 52% of the market share (approximately 13.5 million condoms) may have a range of product quality defects that could increase the risk to the end user. Although the sampling of brands 1–8 was weighted towards suspect product in the market and may inaccurately represent the distribution, the percent level of defects observed for airburst and/or freedom from holes suggest that a high level of condoms (potentially over 1 million) could present a serious risk to the end user. More specifically for this portion of the market, the % defective levels (Fig 1) for airburst (with interquartile range) observed for brands 1–8 project an average of 1.2 million defective condoms (range of 0.2–1.7 million), where 1 million defective condoms (range of 0.01–1.9 million) could be estimated for freedom from holes defects.

Based on these results, la Dirección General de Medicamentos, Alimentos y Productos Sanitarios (DIGEMAPS) del Ministerio de Salud (MS) in the Dominican Republic opened an investigation of poor quality condoms in the market. Condoms have been removed from the market in Santo Domingo, and DIGEMAP was in the process of removing the poor quality condoms from the market in the outer provinces. With the exception of one condom brand, authorities have not been able to assess the organization/individuals that brought the poor quality condoms into Dominican Republic. To prevent future entry of these condoms, internal measures and warnings were ordained, and an epidemiological alert was published and disseminated [28].

DIGEMAPS is committed to enforcing existing condom registration requirements and to establishing a monitoring program to ensure that sub-standard condoms do not enter the Dominican Republic market. USAID will continue to provide technical assistance to continue to assess condom quality, and to train staff in procurement, storage and distribution of condoms and others contraceptive methods.

This investigation highlights the need to intensify product quality monitoring programs in the Dominican Republic and other countries. Global experience suggests the number of countries in which poor quality condoms are entering the market is growing. Recent years have witnessed news reports of the sale of large numbers of counterfeit or poor quality condoms in China, Vietnam, India, the United Kingdom, Australia, Kenya, and Ghana. As sales of poor quality or poorly regulated products grow in an increasingly globalized world, countries will need to develop approaches to improve ongoing monitoring and regulation of product quality.

Although commonly considered in the context of medicines [29], several sources for poor quality products exist concerning performance and labeling, namely poor original manufacturing, deterioration in the field, and falsification of the product. Relatively simple investigations, such as sampling product from pharmacies and grocery stores, allows for the detection of poor quality products in the market. These activities, in addition to strengthening surveillance and regulatory resources (financial and human) are needed to improve monitoring of product quality throughout the supply chain.

## Conclusions

Product quality tests were conducted for male condom samples obtained from the Dominican Republic market. Of the ten brands tested, five brands were observed to have serious levels of defects for freedom from holes and airburst (a measure of physical integrity), including issues with packaging / labeling. Three other brands were compliant for the other quality tests, but demonstrated issues with packaging and labeling. Two of the brands showed no quality issues with any of the evaluations conducted. Overall, the condom samples obtained demonstrated serious product quality problems that would greatly reduce the expected effectiveness against the prevention of HIV (and other STD) transmission and unwanted pregnancies. These results were provided to the regulatory authorities in the Dominican Republic for further investigation.
